# Why do we need Cases Journal?

**DOI:** 10.1186/1757-1626-1-1

**Published:** 2008-05-12

**Authors:** Richard Smith

**Affiliations:** 1 BioMed Central, Middlesex House, 34-42 Cleveland Street, London W1T 4LB, UK

To reinvigorate medicine and provide a useful information tool for clinicians and patients

Why would anybody want to publish a journal that is a mountain of case reports? Aren't case reports scientifically discredited? And why in particular would an old stager like me, once editor of the BMJ and strongly associated with evidence-based medicine, want to be the Editor-in-Chief of such a journal [1]? These are questions that I've had to answer to my own satisfaction to take on the job and that I need to answer to your satisfaction if you are ever to access this journal again.

The case for top of the range case reports has already been convincingly made - both by our academic sister publication [2] and by Jan Vandenbroucke, one of the wisest heads in medical science [3,4]. By definition every new condition - whether it s AIDS, SARS, or the next emergent disease - begins with a single case. Case reports have always been important for detecting adverse drug reactions [5,6]. They also matter in understanding mechanism of disease and for recognising rare manifestations of diseases. Jeff Aronson and others have described circumstances where case reports can provide definitive not just indicative evidence [7,8].

But what is the case for mass publication of case reports such as this journal hopes to achieve? And we will publish any case report that is authentic, understandable, and ethical. It might be an account of a cold sore or, as on this launch, a description of my "Beijing cough" that includes no health professional and no treatment. Our radical contention - which is perhaps not so radical to medical teachers - is that every case is important. And we don't have to destroy trees: we can luxuriate in the infinity of cyberspace - and readers can search our database for whatever they fancy.

Health care is in some ways nothing more than an accumulation of case reports just as a population is a collection of people. And just as every person is important and different so is every case - even when "it's just another sore throat". Indeed, part of the idea is to get away from seeing a morning surgery in general practice or an afternoon's list of men undergoing vasectomy as drudgery or factory work. Clinicians have the privilege of dealing with individuals in all their complexity and magnificence. Every person, every "case" can teach us something. We challenge contributors to draw out the lessons - but they don't have to succeed in order to be published because readers may see what they can't and there will be value in the mass that may not be immediately apparent in the single case.

Perhaps some of our contributors will attempt to write case histories like those of Sigmund Freud. To catch the full mystery, excitement, and interest of a case may demand literary skills, and we know that many clinicians and even more patients have such skills. Jean Cocteau asked his doctor why he continued to attend him day and night at his home and the answer was that the doctors learnt from Cocteau because of his ability to describe his symptoms [9]. Cocteau produced a quote that is directly relevant to this endeavour: "Take a commonplace, clean it and polish it, light it so that it produces the same effect of youth and freshness and originality and spontaneity as it did originally, and you have done a poet's job. The rest is literature".

A mass collection of case reports can have practical value for both practitioners and policy makers. The BEACH (Bettering the Evaluation And Care of Health) programme in Australia has used 100 consecutive case reports from a random sample of general practitioners drawn each year to describe "the characteristics of GPs and the patients who consult them, patient reasons for encounter, the problems managed and management techniques used" [10]. It has gone on now for 10 years and so can plot changes in practice. The programme has been very important in developing general practice in Australia.

But this publication will not be a random sample. It will be accumulation of cases. So can they be of any use clinically? Let us consider the case of a doctor faced with a 75-year-old man who is a smoker, had an acute myocardial infarction five years ago, and has chronic obstructive lung disease, arthritis, and depression. Such a combination is not unusual: 40% of patients with long-term conditions have more than one condition, and many have three or more [11]. As Sir Michael Rawlins, chair of the National Institute of Health and Clinical Excellence, showed at a workshop two years ago, follow the NICE guidelines for each condition and the patient will end up in a mess - because NICE guidelines don't deal with comorbidities. More profoundly, they probably never can: the guidelines are based on trials that deliberately excluded patients with comorbidites. You can never do randomised trials on every kind of patient - the patients that GPs meet every day of the week.

What is the answer? GPs might, and will, use their experience - as they have done for centuries. Their experience might be supplemented with evidence from high quality databases that follow every one of a cohort of patients [12]. But they might also search our journal and database to find a patient just like theirs and see how the patient was treated and what happened to him or her (follow up will be very important for our database - see accompanying editorial [13]). Or they might ask in our journal whether anybody has evidence on such a patient. But - an evidence-based purist might argue - this isn't unbiased information: such information is scientifically uninterpretable. The crucial question, however, is whether it's better than nothing. I believe it is. Nick Wald and Joan Morris have argued the case for teleoanalysis - combining evidence of different types - in addition to meta-analysis [14]. Meanwhile, Alejandro R Jadad and Murray W Enkin, two of the great advocates of randomised trials, have written: "Our main wish, from which all others stem, is that RCTs be taken off their pedestal, their exalted position at the top of an artificial evidence hierarchy, that all forms of evidence be appreciated for what they can offer" [15].

Case reports have something to offer. Plus a combination of being able to ask questions plus open access for all gives us a route the "wisdom of crowds". James Surowiecki shows convincingly in his book of that name how the many can be much smarter than the few [16]. He begins his book with the story - or case report - of Francis Galton, one of the fathers of statistics and an enthusiast for eugenics, studying the results of a guess the weight of the ox competition at a country fair - expecting to find evidence that superior people were best at guessing the weight. He didn't find that. What he did find was that the average of the guesses of the whole crowd came astonishingly close. The crowd is smarter than the individuals within it. Crowds can, of course, be dramatically wrong - for example, in knowing the world to be flat - and it's important how we capture the wisdom of crowds.

Ben Shneiderman has written recently in *Science *magazine about the need for what he calls Science 2.0 [17]. Science 1.0 of hypothesis generation and testing remains important, but Science 2.0 will help us understand "challenges [that] cannot be studied adequately in laboratory conditions because controlled experiments do not capture the rich context of Web 2.0 collaboration, where the interaction among variables undermines the validity of reductionist methods". Reductionist methods struggle with the problem of comorbidities and fail to recognise that patients are much more than specimens with a collection of disease but rather complex individuals with their own values. Ours is, I think, a Science 2.0 experiment.

There are then more frivolous - but still important - reasons for encouraging mass publication of case reports. Doctors - just like everybody else - relate more to stories than they do to statistics and abstractions. Successful politicians understand this well - eschewing complicated numbers for powerful human stories. And this is the age of Web 2.0 and Facebook. We are fed up listening to experts and watching the same old television channels. We want to create our own material, live in a bottom up rather than a top down world. That's perhaps why our sister journal and Sermo, "a practicing [online] community of 50,000 physicians [in the US] who exchange clinical insights, observations, and review cases in realtime - all the time", are doing so well [18]. Here on our site everybody who sees a patient - and everybody who is a patient (that's everybody) - can contribute. We urge you to do so. Let's see if we can create something exciting, special, new, fun, and useful.

*I'm grateful to the many people who commented on drafts of this editorial, but I'm painfully conscious that I haven't responded to all of their comments. In fact I've ignored most of them - not because they were silly or wrong but rather that this piece is my silliness and wrongness and because writing in a crowd is hard. Wisdom will come not from endlessly refining this editorial but rather from debating its strengths and weaknesses. I encourage those whom I've ignored to respond*.
